# Vasomotor and Ocular Phenomena in Relation to Rheumatoid Arthritis

**Published:** 1902-12

**Authors:** R. Llewelyn Jones

**Affiliations:** Late Resident Medical Officer, Royal Mineral Water Hospital, Bath


### VASOMOTOR AND OCULAR PHENOMENA IN RELATION TO RHEUMATOID ARTHRITIS.

BY

R. Llewelyn Jones, M.B. Lond., M.R.C.S., L.R.C.P.,

*Late Resident Medical Officer, Royal Mineral Water Hospital, Bath.*

The vasomotor disturbances that occur in rheumatoid arthritis were ably described by Dr. Kent Spender, to whose facile pen we are indebted for a very vivid and detailed description of the varied phenomena. Anyone who has had the opportunity of coming into contact with a large number of such cases must have been struck by the frequency with which the patients complain of dead-white, or blue fingers. It did not at first strike me that these symptoms of local syncope and asphyxia were identical in nature with those occurring in cases avowedly of the type Raynaud described, but the consideration of a large number convinced me that I could not differentiate them.

Raynaud, and later Galezowski, pointed out that temporary alterations occurred in the circulation of the fundus oculi, and during the past twelve months I examined the fields of vision of over fifty cases of rheumatoid arthritis with coincident symptoms of Raynaud's disease. The fields of vision in all were contracted. They presented the following characteristics: Concentric retraction; the retraction, in some cases amounting almost to extinction, and in others less marked but notable; no marked disparity, as a rule, in degree of affection of either nasal or temporal halves. Homonymous hemianopsia of a fleeting character may be found. Optic atrophy is in some cases unmistakable, in others the colouration is suggestive of an earlier stage. During a paroxysm of local syncope or asphyxia occurring in the extremities, Mr. Beaumont and myself observed the changes in the retinal circulation described by Raynaud and Galezowski, *i.e.*, definite narrowing of the central artery and its branches; very marked pulsations in the veins, moniliform in character; and I have seen a curious condition in the vitreous, as if one were looking through a gauzy shimmering film.

In order to substantiate the vasomotor nature of these circulatory changes, fields were taken before and after nitrite of amyl. Expansion followed, but in no case did it reach the periphery, hence in each field there were, if one may say so, three circles—the one previous to nitrite of amyl inhalation, the larger one subsequent, finally the normal field; the middle or functional area, transient, due to the unlocking of the vessels by the nitrite; the outermost area, the one of organic change, which has lost its sensitivity to light.

As illustrative of the fact that functional disorders if long continued tend to pass into the region of organic change, the changes above described form a good example.

The subjective visual sensations in these attacks are interesting, and I cannot do better than use the language of one of my patients, a collier, in describing them. "I cannot get a straight glimpse of anything—things glitter, appear double, and jump. I catch sight at one moment and next they are all in a muddle." The phenomena often came on at meal times and occasioned him no little annoyance. "If an attack comes on at my tea, and I look at the teapot or anything, it becomes blurred and stretches out and doubles until it is all teapots, and rubbing my eyes a'int no use."

As further evidence that these attacks end in organic retinal change, may I state that many fields were taken in the inter-paroxysmal periods and preserved their characteristics when taken some weeks afterwards? As evidence of the continuity of the process with that occurring in the skin, may I cite the fact that they often occur in sequence to a similar attack in the face? And as further proof of their identity, I have known several cases in which an attack in the face, including the external ear, was followed by giddiness, staggering, reeling—sometimes a fall, also a "roaring noise" with deafness, and occasionally unconsciousness—in brief, a replica of the symptoms common in Menière's disease. Reasoning by analogy with what occurs in the eye, this may throw some light on the origin of the deafness so common in rheumatoid arthritis.

To return to the ocular symptoms. Is not the occurrence of fleeting hemianopsia suggestive of possible extension to the deeper recesses of the brain, *i.e.* the optic tracts?

Burney Yeo, in his work on *Therapeutics*, speaks of the heart as a visceral joint. Is not the term equally applicable to the organ of sight? In a paper shortly to be published I have drawn attention to the frequency with which its movements, like the joints of the limbs, are liable in rheumatoid arthritis to be affected, *i.e.* ataxy, nystagmus, paresis, and even paralysis of its external muscles, not to mention affections of its pupillary movements. In support of my belief that the deeper centres of the brain are subject to the caprices of circulation, may I mention that both Mr. Beaumont and myself have observed the phenomena of "alternating ptosis"? This is (so Aldren Turner says) diagnostic of nuclear affection of cranial nerves. Is it not possible that these transient affections depend upon transient circulatory disturbances affecting the nutrition, and consequently the functioning of the nerve centres? Reasoning by analogy, is it not pertinent that such transient ptoses are common in syphilis, which has such a marked predilection for vascular tissues? Is not the occurrence of episcleritis in rheumatoid arthritis another plea for the justice of the term "visceral joint" as applied to the eye?

In support of my contention that the periarticular swellings of rheumatoid owe their origin to similar vasomotor aberrations, may I adduce the following facts:—(1) A condition of local syncope, &c., in one finger often precedes the formation of spindle swellings in that same digit. I have known the sequence occur in three weeks. (2) The arthrodial swellings and the vasomotor phenomena tend to rise and wane concomitantly. In evidence of the condition being spinal in origin, may I state that—(1) The local asphyxia, when it spreads from the periphery to the trunk, tends to follow the paths of spinal rather than peripheral nerve distribution. (2) The deep reflexes rise and wane concomitantly with the joint trouble—with this qualification, that the increased myotatic irritability tends to persist between paroxysms of joint trouble—and if we grant allegiance to the toxæmic origin of rheumatoid, we should expect the delicate reflex mechanisms to respond to lighter degress of toxæmia than are adequate to produce the graver phenomena. The constant recurrence of local asphyxia in the limbs leads, as in the retina, to trophic changes; and in this connection I would draw attention to the frequency with which such sequelæ are found on the outer side of the leg from knee to ankle, *i.e.* skin glossy ivory-white, devoid of hairs, diminution of sweat, disturbed sensation. It is remarkable how accurately it plots out the $L_5$ area of Head, and similar changes may be observed in the upper limb, more commonly over the dorsum of hand and forearm.

The fact that scleroderma occurs in association with rheumatoid arthritis and Raynaud's disease is also suggestive.

The muscular changes in rheumatoid arthritis are also interesting. The similarity of the cramps and spasms of early rheumatoid to those occurring in tetany is notable some time ago, and in this connection may I adduce a striking symptom, *i.e.* the presence of Chvostek's symptom in rheumatoid? I have elicited it in the case of the facial, ulnar, median nerves, &c.

Another similarity is found in the fact that transient obscurations of vision occur in tetany, and also periarticular swellings. I would advert also to the evanescent swelling of muscles that occur, and coincide with exacerbation in neighbouring joints. The bicipital muscles show this well in affections of the elbow. The later atrophy is not, I think, that of disuse. It may be quite marked when there is no fixation; furthermore, spinally related muscles, though remote, also suffer coincidently.

The fact that rheumatoid and Graves's disease occur frequently in one and the same patient is, I think, very interesting, because of the marked vascular characteristics of the latter. I have often thought that here might be found a means of reconciling the conflicting statements with regard to the quickened pulse-rate in rheumatoid. In an attempt to prove that these diseases possibly in addition to clinical similitude have a common bond of causality as regards their origin, I would enlist any etiological factors common to them. Dilatation of stomach and gastric ulcer are known antecedents of tetany. Bouchard states the former is common in rheumatoid. I have seen several cases supervene on peptic ulcer. Tetany occurs after infectious diseases. Rheumatoid is very common after influenza "sore throats," and a few give a history of pneumonia. Tetany is commonly associated with lactation and pregnancy; and the same may be said of rheumatoid, in that it often comes on after confinements. I have known it come on during pregnancy. I have before stated that Graves's disease and rheumatoid arthritis are often associated, and in this connection the relation of tetany to the thyroid gland is instructive. Tetany follows extirpation of the thyroid gland. Several cases of tetany have occurred in pregnancy in women with atrophy of thyroid.

I have seen the following sequence of events in one patient: Exophthalmic goitre, the thyroid swelling diminished, Raynaud symptoms supervened, and now she has rheumatoid arthritis. Her pulse-rate is 120 per minute, and she has tremor and proptosis. This is no isolated instance. It is far from uncommon to find the three conditions—*i.e.*, rheumatoid arthritis, Raynaud's disease, and Graves's disease—associated in one and the same individual.

The space at my disposal forbids my entering more into detail, but I cannot refrain from suggesting that in the cases with apparently atrophied thyroids, and possibly in others, some benefit might accrue from thyroid administration. The fact that Dr. Lodge ameliorated Raynaud symptoms by administering supra-renal extract is also deserving of thought, considering the frequency of concurrent rheumatoid and Raynaud; likewise an employment of lavage of the stomach, if indications point to it as the primary source of the toxæmia.

I would record my grateful thanks to the honorary physicians of the Royal Mineral Water Hospital, and especially to Mr. W. M. Beaumont, as regards the eye phenomena.

